# PAG Masked Protective Physical Exercise-Induced High H_2_S Levels in 5/6 Nephrectomized Rats

**DOI:** 10.5812/ijpr-145620

**Published:** 2024-05-11

**Authors:** Behjat Seifi, Mehri Kadkhodaei, Enayatollah Bakhshi, Abdollah Sajedizadeh, Mina Ranjbaran, Mahdi Hajiaqaei

**Affiliations:** 1 Department of Physiology, Faculty of Medicine, Tehran University of Medical Sciences, Tehran, Iran; 2 Department of Statistics and Computer, University of Social Welfare and Rehabilitation Sciences, Tehran, Iran

**Keywords:** 5/6 Nephrectomy, Exercise, Hydrogen Sulfide, PAG, Hypertension, RSNA

## Abstract

**Background:**

To investigate the mechanisms of exercise therapeutics in preclinical animal models of chronic kidney disease (CKD), PAG (D, L-propargylglycine), an inhibitor of hydrogen sulfide production, was used to examine the protective effects of physical activity on oxidative stress and inflammation levels during CKD.

**Methods:**

Male Wistar rats with CKD, induced by the 5/6 nephrectomy procedure and subjected to 8 weeks of exercise training, received injections of PAG, a cystathionine γ-lyase (CSE) inhibitor, at a dose of 19 mg/kg, i.p., twice a week during those 8 weeks. The systolic blood pressure (BP) and renal sympathetic nerve activity (RSNA) were assessed. Additionally, plasma creatinine, BUN, renal hydrogen sulfide (H_2_S) levels, oxidative stress, and inflammatory markers were evaluated.

**Results:**

In the PAG group, inhibition of H_2_S production significantly reversed the improvements in plasma creatinine, BUN, renal malondialdehyde (MDA) level, superoxide dismutase (SOD) activity, TNF-α, and IL-6 that were achieved by exercise. Additionally, high RSNA and high BP, which were also reversed in the PAG group, compared to the CKD group subjected to exercise training.

**Conclusions:**

The results suggest that the improvement in BP, oxidative stress, and inflammation status by exercise in CKD may be at least partially due to CSE/H_2_S signaling.

## 1. Background

Hydrogen sulfide (H2S) functions as a signaling gas molecule (1) and plays essential roles in various biological processes. Studies on hypertensive and ovariectomized rats have shown that exercise increases the levels of hydrogen sulfide in the endothelial cells of the heart and aorta, as well as the expression of the cystathionine γ-lyase (CSE) gene, which is crucial for hydrogen sulfide production. In mammals, H_2_S is produced by three key enzymes: Cystathionine β-synthase (CBS), CSE, and 3-mercaptopyruvate sulfur transferase (MST), with CSE being the primary enzyme in the kidney, liver, vascular smooth muscle cells, and endothelial cells. It has been shown that after kidney transplantation, renal CSE correlates with kidney function but not CBS. Hydrogen sulfide levels may decrease in chronic renal disease (2). Our observations indicate that long-term NaHS treatment effectively prevents the progression of chronic kidney disease (CKD) by enhancing the balance between oxidants and antioxidants, and by reducing inflammation and apoptosis (3). In the kidneys, H_2_S regulates tubular reabsorption of sodium, vasodilation, and glomerular filtration rate (GFR) (4). During hypoxic conditions, H_2_S accumulates in the renal medulla, which leads to increased blood volume. Additionally, H_2_S improves renal medulla O_2_ supply by directly inhibiting mitochondrial respiration and dilating the descending vasa recta (5). Given that medullary hypoxia is a common consequence of CKD (6), a deficiency in H_2_S may impair these important adaptive mechanisms.

Clinical evidence suggests a link between decreased physical activity and deteriorating renal function, underscoring the importance of exploring evidence-supported therapeutic options that focus on nutrition and physical exercise (7). A recent study examined the association between adherence to physical activity guidelines for aerobic activity and sedentary time, and their effects on mortality and disease progression among US adults with CKD. It found that increased aerobic activity and reduced sedentary time were associated with decreased mortality and prevention of disease progression (8). Additionally, resistance exercise training has been shown to increase muscle size and strength. Given the compromised physical function of patients with CKD, even modest increases in exercise capacity or strength can lead to significant improvements in functional status. Furthermore, physical activity may enhance survival rates or potentially slow the progression of CKD (9).

In a previous study conducted in our lab, a significant increase in renal H_2_S levels was demonstrated following long-term exercise in CKD animals (10). In the present study, the CSE enzyme and production of H_2_S were inhibited by D, L-propargylglycine (PAG).

## 2. Objectives

Therefore, this study aimed to evaluate the hypothesis that inhibiting H_2_S production via CSE enzyme inhibition could mask the protective effects of physical exercise on renal damage, hypertension, and renal sympathetic nervous activity in preclinical animal models of CKD.

## 3. Methods

Male Wistar rats weighing 220 - 250 g were obtained from the Physiology Animal House. This study received ethical approval from the Ethical Committee of Experimental Animals of Tehran University of Medical Sciences under the approval code IR.TUMS.MEDICINE.REC.1395.638.

Rats were randomly assigned to four groups, with six in each: 1- Sham, 2- CKD, 3- Exercise (Exe), 4- PAG. The induction of CKD was performed using the 5/6 nephrectomy method (10). Three weeks post-surgery, the animals began exercising on a treadmill starting at a speed of 10 meters per minute for one week, followed by an increase to 18 meters per minute for an additional eight weeks. In the PAG group, animals received an injection of the CSE enzyme inhibitor (PAG, 19 mg/kg, intraperitoneally, twice a week) for eight weeks.

A recent recommendation involves the measurement of out-of-office blood pressure (BP) to confirm the diagnosis of hypertension (11). Therefore, in two animals from each group, a telemetry transmitter was inserted into the abdominal aorta for continuous arterial BP recording during the last two days of the experiments. At the end of the twelfth week, left renal sympathetic nerve activity (RSNA) was recorded for two hours. In the remaining animals of each group, systolic BP was measured using the Power Lab Tail cuff system.

At the conclusion of the study, blood and kidney tissue samples were collected to evaluate markers of oxidative stress (levels of MDA and activity of SOD) and inflammatory indices (TNF-α and IL-6 levels).

### 3.1. Blood Pressure Transmitter Remote Installation Method

Two days before the end of the experiments, rats were anesthetized with isoflurane (5%, maintained at a dose of 1 - 3%). The abdomen of each rat was opened to locate the abdominal aorta below the renal arteries within the peritoneal cavity. A catheter transmitter (TRM54p) was then inserted into the aorta and advanced up to the renal arteries. The catheter was secured to the aorta using tissue glue. After the glue dried, the transmitter device was positioned right in front of the animal's abdomen, ensuring it made contact with the ground. Surgical mesh around the transmitter was sutured to the subcutaneous layer. The skin and other layers were then sutured closed. Analgesic and antibiotic drugs were administered intramuscularly. Subsequently, each animal was placed separately on a pad (SmartPad, TR180) connected to a Configurator (TR190) for continuous recording of BP and heart rate. The PowerLab system and LabChart software recorded the signals. Blood pressure was recorded for 10 seconds every 2 minutes at a frequency of 500 Hz over those two days.

### 3.2. Renal Sympathetic Nerve Activity Recording Method

To record the activity of the renal sympathetic nerve, on the final day of the experiments, the left renal nerve was exposed using an optical microscope. The nerve was positioned on a pair of silver electrodes. Once an acceptable signal-to-noise ratio was achieved, vaseline oil was applied to cover both the nerve and the electrodes. Nerve signals were then amplified by an AC/DC amplifier with low and high frequency cutoffs set at 30 - 3000 Hz. Amplified signals were integrated with a time constant of 20 seconds. The PowerLab data acquisition system was used to concurrently record both raw and integrated RSNA.

### 3.3. Inflammatory Indices Assessments

Tumor necrosis factor-alpha (TNF-α) and interleukin-6 (IL-6) concentrations were measured in plasma samples using ELISA.

### 3.4. Renal Oxidative Stress Assessments

Malondialdehyde (MDA) levels and superoxide dismutase (SOD) activity were measured in the supernatant of kidney tissue homogenates. Malondialdehyde levels were assessed according to the method described by Esterbauer and Cheeseman (12). The technique developed by Paoletti and Mocali was used to measure SOD activity (13).

### 3.5. Measurement of Renal Hydrogen Sulfide Levels

Hydrogen sulfide concentration in the kidney tissues was measured using methods previously described (14).

### 3.6. Statistical Analysis

Data are expressed as mean ± SEM. One-way ANOVA followed by a post hoc Tukey's test was used. The significance level was set at P < 0.05.

## 4. Results

### 4.1. Effect of PAG on the Beneficial Effects of the Exercise

**Figure 1.**
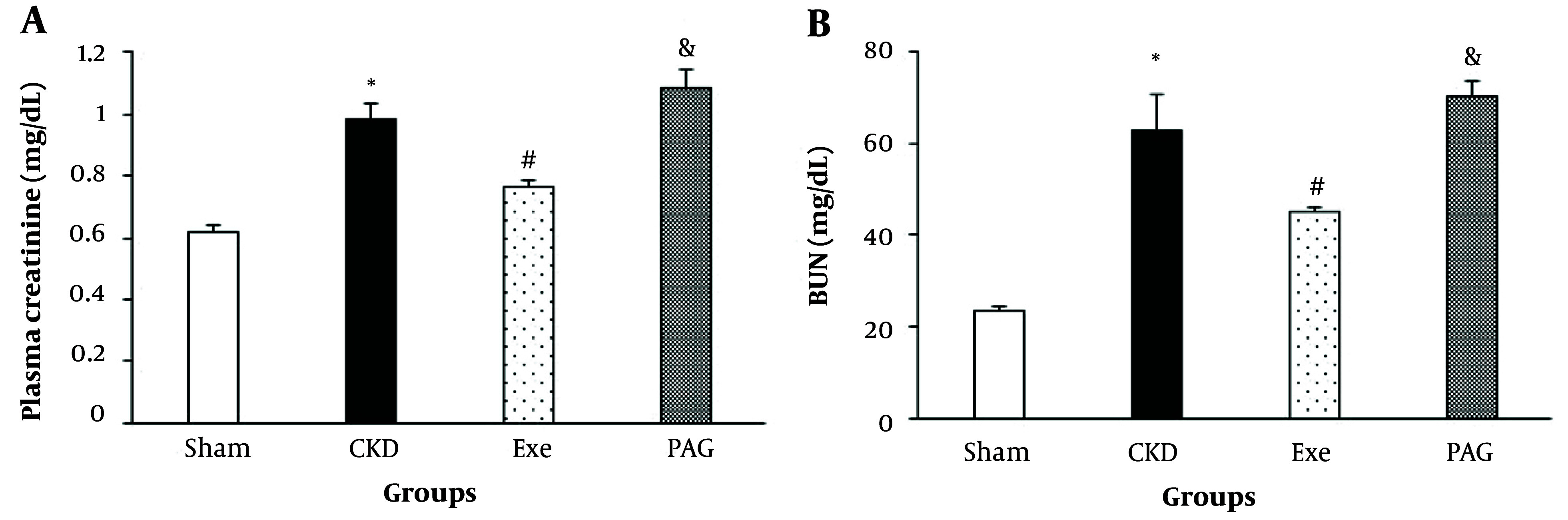
A, changes in plasma creatinine; and B, BUN in different groups. Data are presented as Mean ± SEM. Chronic kidney disease (CKD), 5/6 Nx; Exe, 5/6 Nx + exercise; PAG, 5/6 Nx + exercise + D L-propargylglycine.* P < 0.05 significance differences with Sham group. # P < 0.05 significance differences with CKD group. & P < 0.05 significance differences with Exe group.

Eight weeks of PAG administration along with physical exercise resulted in significantly higher plasma creatinine and BUN levels compared to the exercise-only group (Figure 1, P < 0.05).

**Figure 2.**
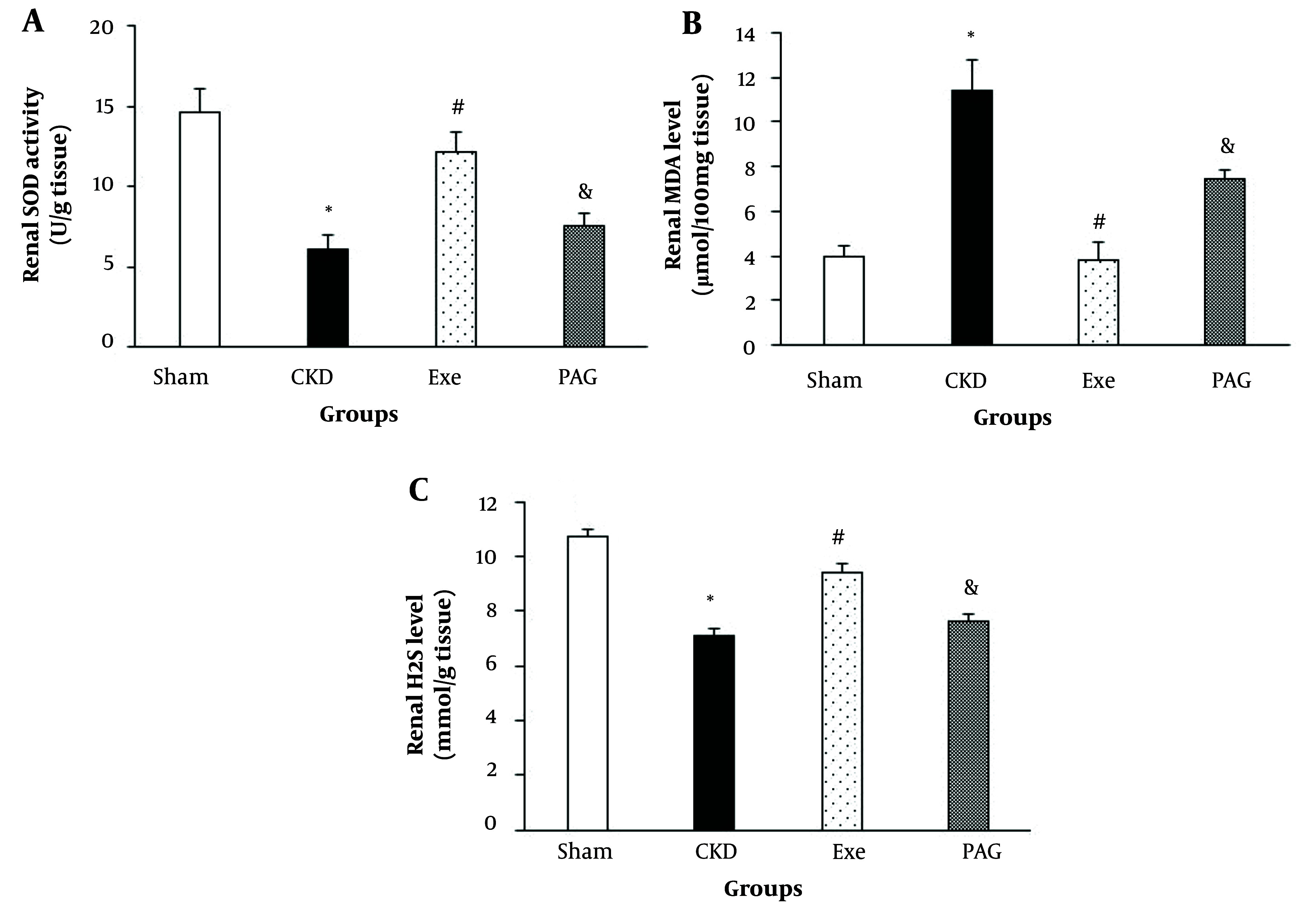
A, changes in renal superoxide dismutase (SOD) activity; B, malondialdehyde (MDA) level and hydrogen sulfide (H_2_S) level; C, in different groups. Data are presented as Mean ± SEM. Chronic kidney disease (CKD), 5/6 Nx; Exe, 5/6 Nx + exercise; PAG, 5/6 Nx+exercise+D, L-propargylglycine.* P < 0.05 significance differences with Sham group. # P < 0.05 significance differences with CKD group. & P < 0.05 significance differences with Exe group.

PAG administration combined with physical exercise led to significantly lower renal SOD activity and MDA levels compared to the exercise-only group (Figure 2, P < 0.05).

Additionally, PAG administration along with physical exercise resulted in a significantly lower renal H_2_S level compared to the exercise-only group (Figure 2, P < 0.05).

**Figure 3.**
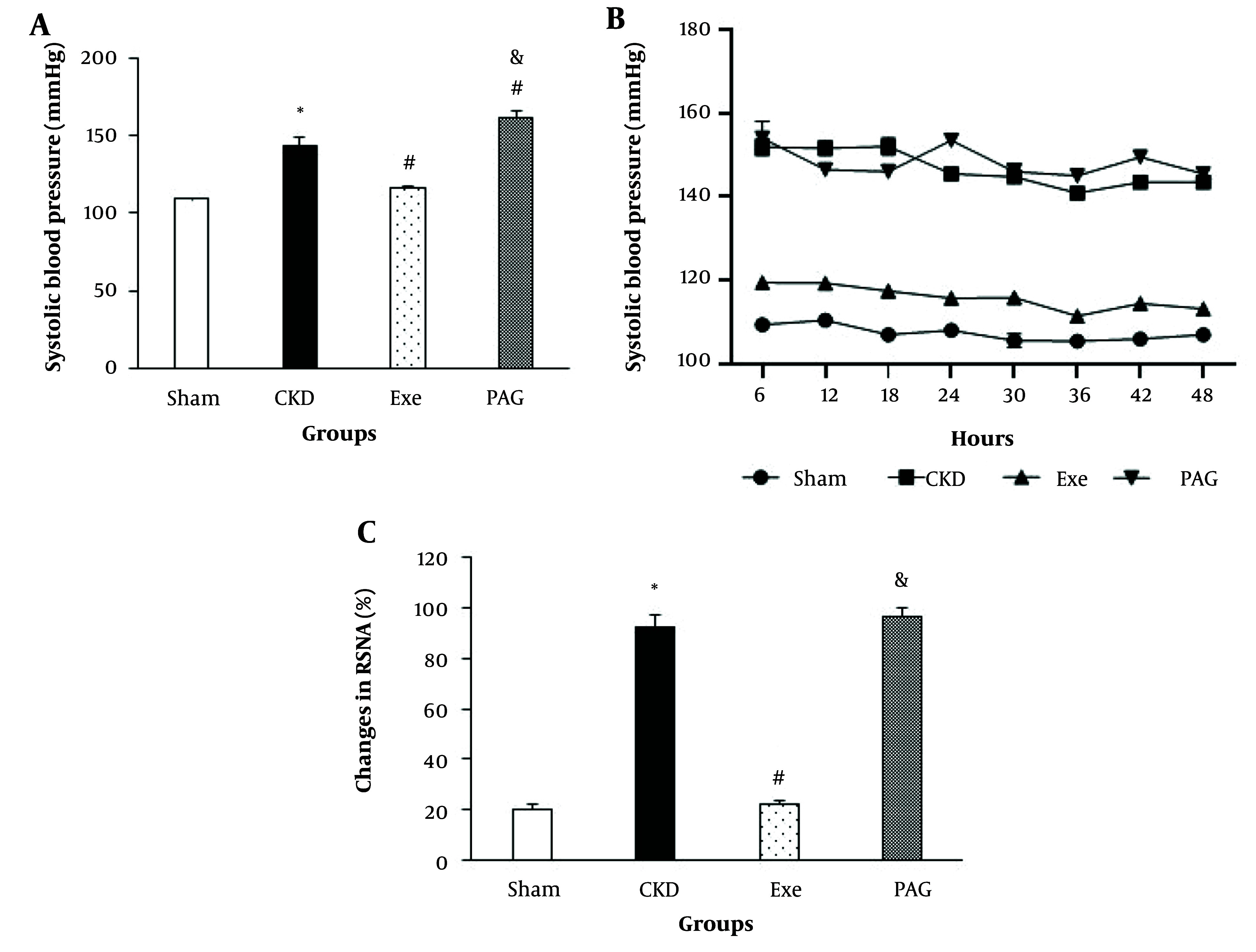
A, changes in systolic blood pressure (BP) in the end of 8 weeks; B, SBP monitoring for the last 48 hours; and C, renal sympathetic nerve activity (RSNA) in different groups. Data are presented as Mean ± SEM. Chronic kidney disease (CKD), 5/6 Nx; Exe, 5/6 Nx+exercise; PAG, 5/6 Nx + exercise + D, L-propargylglycine.* P < 0.05 significance differences with Sham group. # P < 0.05 significance differences with CKD group. & P < 0.05 significance differences with Exe group.

PAG administration combined with physical exercise also resulted in significantly higher systolic BP and RSNA compared to the exercise-only group (Figure 3A and C , P < 0.05).

Telemetry BP monitoring was performed in two rats to further characterize the effects of PAG on the benefits of exercise training on systolic arterial BP during CKD-induced hypertension. Similar to observations made using the tail cuff method, administration of PAG led to an average increase of 30 - 40 mmHg in systolic BP over 48 hours of monitoring (Figure 3B). Consistent with the tail-cuff method data, PAG counteracted the protective effects of exercise training against hypertension, such that by the end of the 8-week treatment period, BP had returned nearly to the levels seen in the CKD group.

**Figure 4.**
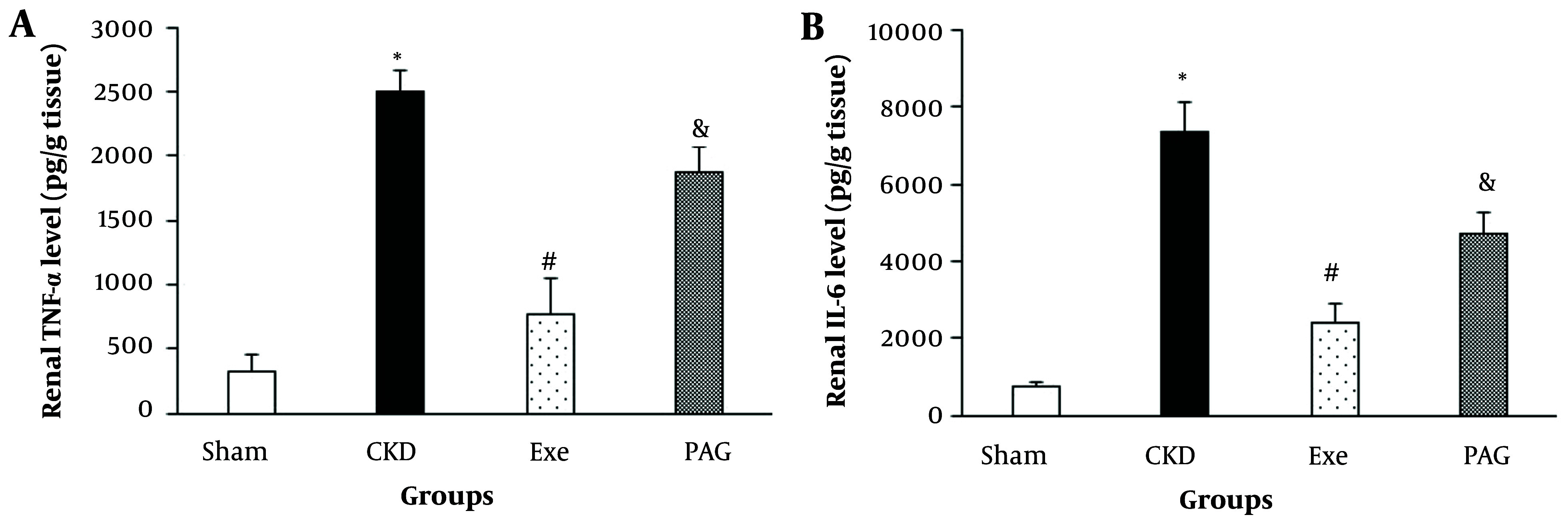
A, Changes in renal TNF-α; B, and IL-6 levels in different groups. Data are presented as Mean ± SEM. Chronic kidney disease (CKD), 5/6 Nx; Exe, 5/6 Nx + exercise; PAG, 5/6 Nx + exercise + D; L-propargylglycine.* P < 0.05 significance differences with Sham group. # P < 0.05 significance differences with CKD group. & P < 0.05 significance differences with Exe group.

Eight weeks of PAG administration along with physical exercise resulted in significantly higher renal TNF-α and IL-6 levels compared to the exercise-only group (Figure 4, P < 0.05).

## 5. Discussion

The main finding of this study was that in the kidneys, inhibition of CSE-mediated H_2_S formation masked the beneficial effects of physical exercise in 5/6 nephrectomized rats. Physical exercise was found to restore renal H_2_S levels. Recent research demonstrated that exercise training increased the expression of CBS/CSE and enhanced endogenous H_2_S production in the renal tissues of diabetic nephropathy (15). Additionally, it was observed that both H_2_S and the expression of Nrf2-related antioxidant pathways increased following exercise training. However, blockade of CSE by PAG in this study resulted in lower H_2_S levels, which deteriorated renal function. Consistent with this, Bos et al. reported that CSE deficiency was associated with increased mortality, and the administration of exogenous H_2_S rescued mice from ischemic injury and eventual death (16). In this study, physical exercise increased renal H_2_S levels and SOD activity, while decreasing MDA production and TNF-α and IL-6 levels in the remnant kidney tissues. Inflammatory cytokines play significant roles in the development and progression of CKD (17). Our data similarly indicated that cytokine levels were significantly higher and that PAG masked the positive effects of exercise on oxidative stress and inflammation. Given that physical exercise resulted in increases in renal H_2_S levels, we suggest that these positive effects may be a result of an increase in renal CSE activities.

It is well known that H_2_S decreases BP in experimental models of hypertension (18). A deficiency in H_2_S-producing enzymes leads to high BP, while the use of H_2_S donors reduces BP and provides protection against organ damage. Since H_2_S may increase the sensitivity of carotid sinus baroreflexes (19), it has been demonstrated that exogenous H_2_S inhibits sympathetic outflow in this area (20). The results of the present study suggest that the high H_2_S levels induced by physical exercise may decrease BP by reducing sympathetic activation through the reduction of oxidative stress and inflammation. Treatment of hypertensive rats with thiosulfate resulted in lower BP and decreased organ damage (21). Oxidative stress and inflammatory cytokines are implicated as important factors in triggering increased sympathetic activity and the development of high BP (22). During hypertension, renal denervation has proven to be effective in reducing BP. Sensory information from the kidney is integrated at the level of pre-autonomic neurons within the hypothalamic nuclei (23). It is possible that in CKD, tonic activation of the central chemo-reflex pathways may impair this reflex, leading to further increases in RSNA. It was demonstrated that abnormally muscle reflex-evoked increases in sympathetic activity mediate exaggerations in BP (24).

In the present study, direct recording of BP in freely moving and conscious rats overcame many of the limitations associated with other indirect assessments of BP. In a study with conscious CKD rats, Salman et al. described direct telemetric recording of sympathetic nerve activity (SNA) to the kidney and presented evidence that high BP is significantly correlated with increased RSNA and decreased renal function (25). Increased sympathetic activity directed towards the CKD kidney may play a significant role in the long-term regulation of BP (26). Therefore, during CKD, sympathetic overdrive may contribute to the resistant hypertensive state. Further investigation is needed to clarify how the inhibition of H_2_S production and the use of H_2_S donors can achieve similar outcomes on the protective effects of exercise in CKD.

### 5.1. Conclusions

The data from the present study demonstrate that reduced H_2_S production might be associated with increases in oxidative stress and inflammation, which prevent the protective effects of physical exercise on CKD. Our findings suggest that the high H_2_S level induced by physical exercise may decrease hypertension in CKD by reducing sympathetic activation through the reduction of oxidative stress and inflammation, and this effect might be partially CSE/H_2_S dependent.

## Data Availability

The study's original contributions have been included in the article's content, and the corresponding author can be contacted with further questions.
